# Development and validation of a prediction model for 90-day mortality among critically ill patients with AKI undergoing CRRT

**DOI:** 10.1007/s40620-025-02237-1

**Published:** 2025-03-10

**Authors:** Tingting Wang, Sha Xu, Yufei Yuan, Wenbin Guo, Hongliang Zhang, Jiajun Sun

**Affiliations:** 1 Department of Intensive Care Unit, The Second People’s Hospital of Liaocheng, Linqing, 252600 Shandong Province China; 2 Department of Cardiology, The Second People’s Hospital of Liaocheng, Linqing, 252600 Shandong Province China; 3 Department of Nephrology, The Second People’s Hospital of Liaocheng, Linqing, 252600 Shandong Province China

**Keywords:** Acute kidney injury, Continuous renal replacement therapy, Prediction model, Mortality

## Abstract

**Background:**

Acute kidney injury (AKI) is frequent among intensive care unit (ICU) patients and is linked with high morbidity and mortality. In the absence of specific pharmacological treatments for AKI, continuous renal replacement therapy (CRRT) is a primary treatment option. This study aimed to develop and validate a predictive model for 90-day mortality in critically ill patients with AKI undergoing CRRT.

**Methods:**

Clinical data from DATADRYAD were used. We randomly divided 1121 adult patients receiving CRRT for AKI into training (80%, *n* = 897) and validation (20%, *n* = 224) cohorts. A nomogram prediction model was developed using Cox proportional hazards regression with the training set, and was validated internally. Model performance was evaluated based on calibration, discrimination, and clinical utility.

**Results:**

The model, incorporating seven predictors—SOFA score, serum creatinine, blood urea nitrogen, albumin levels, Charlson comorbidity index, mean arterial pressure at CRRT initiation, and phosphate levels 24 h after CRRT initiation—demonstrated robust performance. It achieved a C-index of 0.810 in the training set and 0.794 in the validation set.

**Conclusions:**

We developed and validated a predictive model based on seven key clinical predictors, showing excellent performance in identifying high-risk patients for 90-day mortality in AKI patients undergoing CRRT.

**Graphical abstract:**

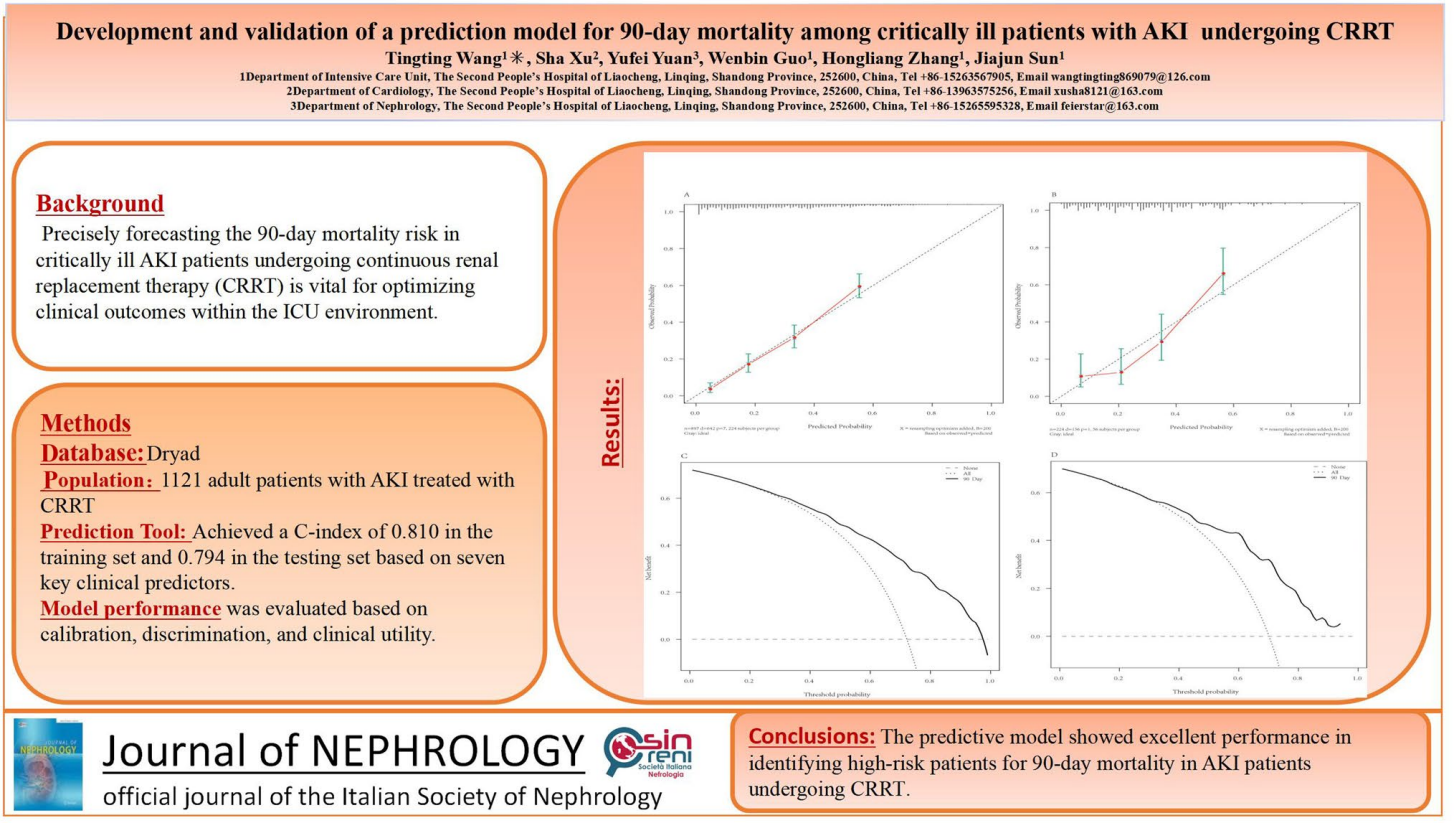

**Supplementary Information:**

The online version contains supplementary material available at 10.1007/s40620-025-02237-1.

## Introduction

Acute kidney injury (AKI) is a global public health concern, associated with significant morbidity, mortality, and healthcare costs [1]. Approximately 50% of intensive care unit (ICU) patients experience AKI [2]. Continuous renal replacement therapy (CRRT) is a primary treatment option for these patients. However, even with appropriate CRRT, morbidity and mortality rates remain alarmingly high [3, 4]. Therefore, early detection and identification of prognostic factors are crucial to improving the prognosis and preventing death in high-risk patients. Additionally, robust mortality risk prediction can enhance shared decision-making between clinicians and patients (and/or their families) during CRRT discussions.

Several tools are used to predict mortality in ICU patients, such as the Acute Physiology and Chronic Health Evaluation II (APACHE II), the Mortality Probability Model II, and the Simplified Acute Physiology Score II (SAPS II) [5–7]. Although various mortality prediction models have been developed [8, 9], they do not specifically target patients requiring CRRT for AKI. Traditional scoring systems like APACHE II and the Sequential Organ Failure Assessment (SOFA) score are generally effective for predicting ICU mortality [10, 11] but fall short in predictive power for AKI patients undergoing CRRT [8]. Therefore, there is a critical need to develop a new scoring model specifically tailored for these patients.

Given that delayed diagnosis significantly contributes to the high mortality rates among AKI patients undergoing CRRT in the ICU, early identification of these high-risk individuals is crucial. Timely recognition allows clinicians to implement effective interventions, thereby reducing mortality and improving overall quality of life. This study aims to develop and validate a user-friendly predictive model for accurately estimating 90-day mortality in AKI patients requiring CRRT, using data from a retrospective cohort. The internal validity of the model was rigorously assessed. By providing more personalized prognoses, this predictive tool is intended to help clinicians and patients make informed treatment and management decisions.

## Methods

### Data source and study population

Based on a retrospective cohort study [12], we accessed the original data from the DRYAD database (10.5061/dryad.6v0j9) and conducted a secondary analysis with a different hypothesis, ensuring no violation of the authors’ rights. Informed consent was waived due to the study's retrospective nature. All patients’ private information and identities were kept confidential in the database. Our findings were reported in accordance with the TRIPOD guidelines [13]. In this study, patients with a recorded time of death at day 90 being 0 (*n* = 23) were excluded when utilizing Cox proportional hazards regression to develop a prediction model. Ultimately, 1121 patients were included in the final analysis and randomly split into training (80%, *n* = 897) and validation (20%, *n* = 224) sets to evaluate the prognostic models. Figure S1 illustrates the patient selection process.

### Study variables

Demographic and clinical data, such as age, sex, body mass index (BMI) at ICU admission, systolic blood pressure (SBP), diastolic blood pressure (DBP), mean arterial pressure (MAP), comorbidities including hypertension (HTN), diabetes mellitus (DM), myocardial infarction (MI), congestive heart failure (CHF), peripheral vascular disease (PVD), chronic obstructive pulmonary disease (COPD), and mechanical ventilation (MV), Charlson comorbidity index (CCI), cause of AKI and CRRT, CRRT dose at CRRT initiation and urine output at 2 h after CRRT initiation (UO_2h) were collected. Biochemical laboratory data at CRRT initiation, including data on hemoglobin (Hb) levels, white blood cell (WBC) counts, and levels of C-reactive protein (CRP), serum creatinine (Cr), blood urea nitrogen (BUN), albumin (ALB), bicarbonate, potassium (K), and serum phosphate at CRRT initiation (P_0 h) and at 24 h after CRRT initiation (P_24 h) were collected. To evaluate disease severity and organ failure, Acute Kidney Injury Network (AKIN) stages, SOFA scores, and APACHE II scores were recorded at CRRT initiation. The primary outcome of this study was 90-day mortality, with the secondary outcome being 28-day mortality.

### Statistical analysis

Eligible patients were randomly divided into training and validation sets at a ratio of 8:2. Data from the training set were used to perform Cox regression analysis and construct the nomogram, whereas data from the validation set were used to validate the model. Continuous variables are expressed as means with standard deviation (SD) or medians with interquartile range (IQR), and categorical variables are presented as counts with percentages. Student’s *t*-test and the Wilcoxon rank-sum test were used to compare continuous variables, and Pearson’s chi-square test was used for categorical variables. Univariate and multivariate Cox regression analyses of potential factors were performed to identify significant predictors. Variables exhibiting a *P*-value < 0.2 in the univariate analysis, and those considered clinically relevant were entered into the multivariate Cox regression model. Following the identification of the associated risk factors using multivariate Cox regression analysis, a predictive nomogram was developed using the BSR method. Internal validation was performed via bootstrap resampling using 200 random samples drawn with replacements. Receiver operating characteristic (ROC) curves with area under the curve (AUC) values were used to determine the discriminative ability. A calibration curve was used to evaluate the agreement between actual and predicted survival probabilities. We performed decision curve analysis to clarify the clinical usefulness of our risk stratification model. Statistical significance for all analyses was set at *P* < 0.05 (2-sided). All analyses were performed using R Statistical Software (http://www.R-project.org, The R Foundation) and Free Statistics software version 1.7.1.

The sample size for the prediction model was determined by ensuring a minimum ratio of 10 [14] between the number of observed events and the number of predictors that could be estimated.

## Results

### Baseline characteristics

Figure S1 shows the recruitment process. Inclusion criteria were met by 1121 patients who were then enrolled into this study. Table 1 summarizes the characteristics of the patients in the training and validation sets. The training and validation sets had 897 (60.8% men) and 224 (64.3% men) patients with an average age of 63.6 ± 14.3 years and 62.1 ± 14.4 years, respectively. The mortality rates at 90 days were 71.6% and 69.6% in the training and validation sets, respectively. No significant differences were observed in clinical characteristics between the two sets. Missing data were imputed using the multiple imputation chain equation method with five datasets. No substantial differences were found in the distribution of missing variables between the participants with observed data and those with imputed data (Table S1). Table S2 summarizes the differences in baseline characteristics between survivors and non-survivors in the two sets. When the patients in the training set were categorized according to 90-day mortality, most baseline variables differed between survivors and non-survivors. Compared with survivors in the two sets, non-survivors were more seriously ill, as indicated by lower ALB, SBP, DBP, and MAP and higher SOFA and APACHE II scores at CRRT initiation.

**Table 1:** Baseline patient characteristics in the training and validation sets

Variables	Training set (*n* = 897)	Validation set (*n* = 224)	*P*-value
Death_90 day, *n* (%)			0.569
Survivors	255 (28.4)	68 (30.4)	
Non-survivors	642 (71.6)	156 (69.6)	
Age (years)	63.6 ± 14.3	62.1 ± 14.4	0.159
Male	545 (60.8)	144 (64.3)	0.332
HTN (%)	476 (53.1)	116 (51.8)	0.731
DM (%)	318 (35.5)	73 (32.6)	0.56
MI (%)	93 (10.4)	18 (8)	0.296
HF (%)	154 (17.2)	32 (14.3)	0.3
CVD (%)	91 (10.1)	20 (8.9)	0.646
COPD (%)	63 (7)	16 (7.1)	0.95
MV (%)	698 (77.8)	179 (79.9)	0.497
CCI	3.0 (2.0, 5.0)	3.0 (2.0, 5.0)	0.757
Cause of AKI			0.613
Sepsis	634 (70.7)	156 (69.6)	
Nephrotoxin	29 (3.2)	6 (2.7)	
Ischemia	76 (8.5)	15 (6.7)	
Surgery	74 (8.2)	19 (8.5)	
Others	84 (9.4)	28 (12.5)	
Cause of CRRT			0.411
Volume overload (%)	126 (14)	28 (12.5)	
Metabolic acidosis (%)	193 (21.5)	44 (19.6)	
Hyperkalemia (%)	48 (5.4)	7 (3.1)	
Uremia (%)	84 (9.4)	29 (12.9)	
Oliguria (%)	233 (26)	58 (25.9)	
Others (%)	213 (23.7)	58 (25.9)	
AKIN stages			0.449
Stage 2 (%)	230 (25.6)	63 (28.1)	
Stage 3 (%)	667 (74.4)	161 (71.9)	
BMI (kg/m^2^)	23.8 ± 4.6	23.7 ± 4.1	0.716
SOFA score	12.0 ± 3.6	12.2 ± 3.4	0.531
APACHE II score	27.3 ± 7.9	26.7 ± 8.0	0.252
SBP (mmHg)	111.6 ± 21.1	114.8 ± 19.8	0.044
DBP (mmHg)	60.2 ± 14.2	61.5 ± 14.0	0.207
MAP (mmHg)	77.2 ± 14.5	79.4 ± 14.4	0.039
Hemoglobin (g/dL)	9.6 ± 2.2	9.7 ± 2.2	0.565
WBC (μL)	11,730.0 (6690.0, 18,620.0)	10,660.0 (5622.0, 18,880.0)	0.298
Albumin (g/dL)	2.6 ± 0.6	2.6 ± 0.6	0.899
Potassium (mEq/L)	4.7 ± 1.1	4.5 ± 1.0	0.008
Bicarbonate (mEq/L)	16.9 ± 5.7	17.0 ± 5.7	0.85
BUN (mg/dL)	50.0 (33.0, 74.0)	49.0 (35.0, 72.2)	0.883
Phosphate (mg/dL)	5.8 ± 2.5	5.6 ± 2.1	0.275
Creatinine (mg/dL)	2.7 ± 1.6	2.8 ± 1.9	0.676
CRRT dose (ml/kg)	36.7 ± 4.8	36.5 ± 4.8	0.476
CRP (mg/L)	72.5 (20.4, 168.0)	90.6 (28.0, 186.7)	0.102

*Abbreviations*: HTN, hypertension; DM, diabetes mellitus; MI, myocardial infarction; HF, heart failure; CVD, cerebrovascular disease; COPD, chronic obstructive pulmonary disease; MV, mechanical ventilation; CCI, Charlson Comorbidity Index; AKI, acute kidney injury; CRRT, continuous renal replacement therapy; AKIN, Acute Kidney Injury Network; BMI, body mass index; SOFA, Sequential Organ Failure Assessment; APACHE II, Acute Physiology and Chronic Health Evaluation II; SBP, systolic blood pressure; DBP, diastolic blood pressure; MAP, mean arterial pressure; WBC, white blood cell count; BUN, blood urea nitrogen; CRP, C-reactive protein

### Development of mortality prediction model

**Fig. 1:**
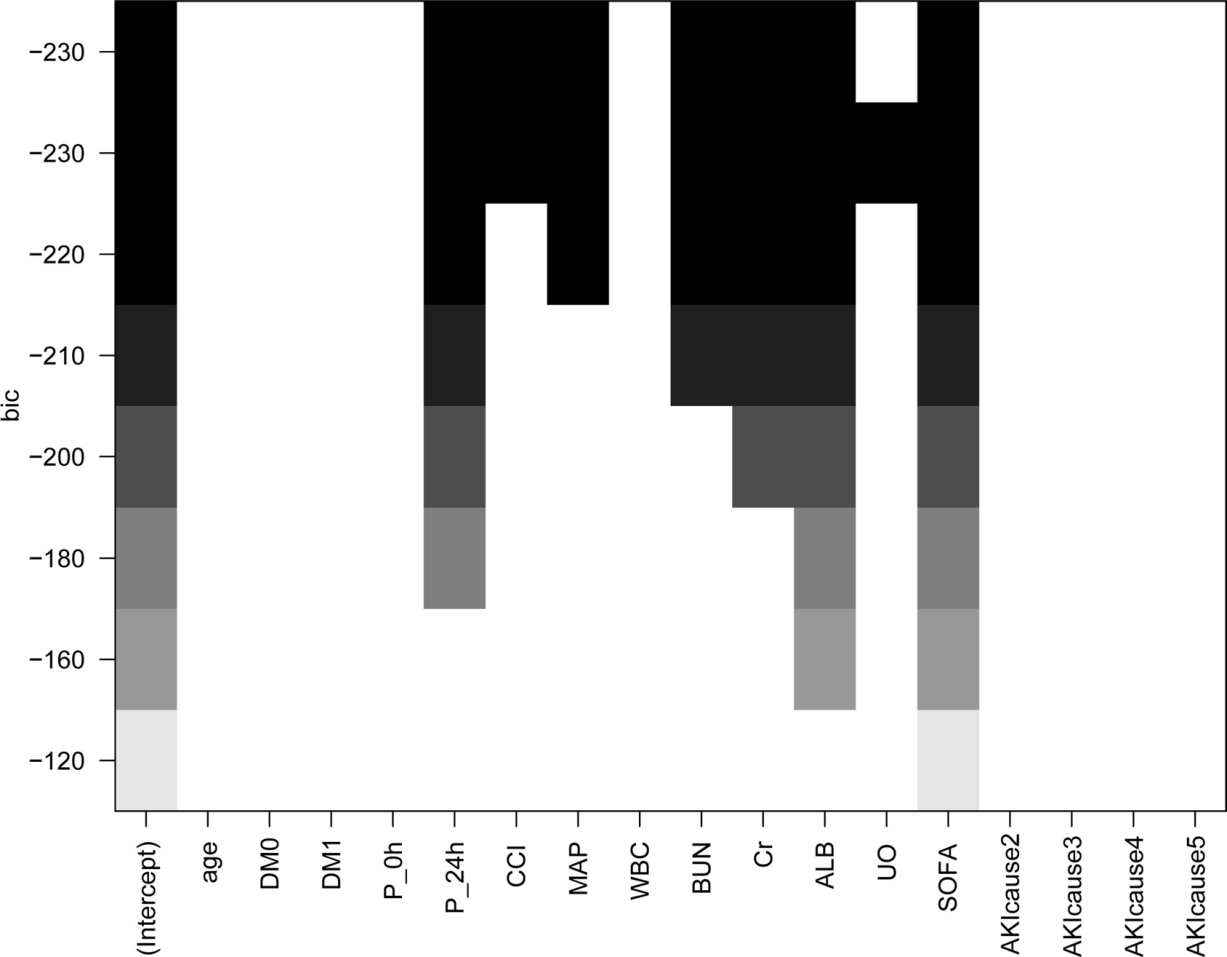
Selection of variables using the BSR method. *Abbreviations*: DM, diabetes mellitus; P_0h, serum phosphate before CRRT, Continuous Renal Replacement Therapy initiation; P_24h, serum phosphate at 24 h after CRRT initiation; CCI, Charlson Comorbidity Index; MAP, mean arterial pressure; WBC, white blood cell count; BUN, blood urea nitrogen; Cr, creatinine; ALB, albumin; UO, urine output at 2 h after CRRT initiation; SOFA, Sequential Organ Failure Assessment score; AKI, acute kidney injury

Basic demographics, vital signs, and laboratory tests in the training set were further examined using univariate and multivariate Cox regression analyses to predict 90 day mortality (Table 2). Variables, encompassing comorbidities such as metabolic acidosis, diabetes mellitus, and hypertension, alongside biochemical markers indicative of disease severity including WBC counts, Cr levels, ALB levels, P_24 h, Hb levels, glomerular filtration rate (GFR), P_0 h, and severity indices such as CCI, APACHE II, SOFA as well as general conditions such as SBP, DBP, MAP, urine output, mechanical ventilation, and BMI were identified as potential predictors of 90-day mortality in the univariate analysis (*p* < 0.05). All candidate factors along with BUN that were considered clinically relevant were entered into a multivariate Cox regression model. The BSR method conferred significant advantages in variable selection, as it exhaustively computed all possible combinations of variables. The final selected combination was determined to be optimal based on the minimum Bayesian Information Criterion (BIC). As illustrated in Fig. 1, the selection process included all 7 parameters, resulting in a minimum Bayesian Information Criterion value of − 230. Consequently, the BSR method led to the selection of different variables for the final model: SOFA score, Cr, BUN, ALB, CCI, and MAP at CRRT initiation; and phosphate levels at 24 h after CRRT initiation (Fig. 1).

**Table 2:** Univariate and multivariate Cox regression analysis of selected clinical features in the training set

Variables	Univariate analysis		Multivariate analysis	
	HR (95%CI)	*P*-value	HR (95%CI)	*P*-value
Age	1.00 (1.00 ~ 1.01)	0.32	1.01 (1.01 ~ 1.02)	** < 0.001**
Female	0.99 (0.84 ~ 1.156)	0.86	1.13 (0.95 ~ 1.35)	0.177
BMI	0.98 (0.96 ~ 1)	0.029	0.99 (0.97 ~ 1.01)	0.27
SBP	0.99 (0.98 ~ 0.99)	< 0.001	0.99 (0.99 ~ 1.00)	**0.015**
DBP	0.993 (0.988 ~ 0.999)	0.016	1.00 (0.99 ~ 1.02)	0.416
MAP	0.99 (0.98 ~ 0.99)	< 0.001	0.99 (0.99 ~ 1.00)	**0.005**
MV	1.64 (1.34 ~ 2.01)	< 0.001	0.80 (0.61 ~ 1.05)	0.102
UO	0.998 (0.997 ~ 0.999)	< 0.001	0.999 (0.998 ~ 0.999)	**0.023**
WBC	0.82 (0.77 ~ 0.88)	< 0.001	0.92 (0.86 ~ 0.98)	**0.007**
BUN	1.002 (0.999 ~ 1.004)	0.14	1.01 (1 ~ 1.01)	** < 0.001**
ALB	0.67 (0.59 ~ 0.76)	< 0.001	0.67 (0.58 ~ 0.77)	** < 0.001**
P_0h	1.06 (1.03 ~ 1.09)	< 0.001	1.14 (0.79 ~ 1.63)	0.488
P_24h	1.16 (1.13 ~ 1.19)	< 0.001	1.15 (1.1 ~ 1.19)	**< 0.001**
Hb	0.95 (0.91 ~ 0.98)	0.006	0.996 (0.957 ~ 1.037)	0.85
GFR	1.004(1.001 ~ 1.007)	0.012	1.000 (0.995 ~ 1.005)	0.995
PreCr	1.01 (1 ~ 1.03)	0.023	0.997 (0.983 ~ 1.010)	0.62
Cr	0.89 (0.84 ~ 0.94)	< 0.001	0.78 (0.71 ~ 0.85)	**< 0.001**
Metabolic acidosis	1.52 (1.16 ~ 1.99)	0.002	1.48 (1.12 ~ 1.96)	**0.006**
DM	0.02 (0.00 ~ 0.13)	< 0.001	0.01 (0.00 ~ 0.07)	**< 0.001**
HTN	0.74 (0.63 ~ 0.86)	< 0.001	0.83 (0.69 ~ 1.01)	0.056
APACHE II	1.04 (1.03 ~ 1.05)	< 0.001	1.02 (1.01 ~ 1.03)	**0.002**
CCI	1.08 (1.04 ~ 1.12)	< 0.001	1.08 (1.04 ~ 1.12)	** < 0.001**
SOFA	1.11 (1.09 ~ 1.14)	< 0.001	1.09 (1.06 ~ 1.12)	** < 0.001**

Note : Bolded indicate statistically significant results

Abbreviations: BMI, body mass index; SBP, systolic blood pressure; DBP, diastolic blood pressure; MAP, mean arterial pressure; MV, mechanical ventilation; UO, urine output at 2 h after CRRT initiation; WBC, white blood cell count; BUN, blood urea nitrogen; ALB, albumin; P_0h, serum phosphate at CRRT initiation; P_24h, serum phosphate at 24 h after CRRT initiation; Hb, hemoglobin; GFR, glomerular filtration rate; PreCr, creatinine level before CRRT initiation; Cr, creatinine; DM, diabetes mellitus; HTN, hypertension; APACHE II, Acute Physiology and Chronic Health Evaluation II; CCI, Charlson Comorbidity Index; SOFA, Sequential Organ Failure Assessment

### Risk prediction nomogram development

**Fig. 2:**
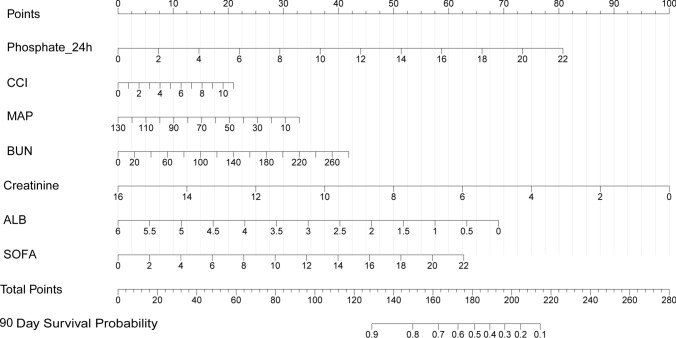
Nomogram to calculate risk score and predict the risk of 90-day mortality. *Abbreviations*: Phosphate_24 h, serum phosphate at 24 h after CRRT, Continuous Renal Replacement Therapy initiation; CCI, Charlson Comorbidity Index; MAP, mean arterial pressure; BUN, blood urea nitrogen; ALB, albumin; SOFA, Sequential Organ Failure Assessment

A prognostic nomogram for 90-day mortality was established using seven prognostic factors obtained from the multivariate Cox proportional hazards model (R^2^ = 0.261, C-index = 0.695) (Fig. 2). The model’s predictive accuracy was further validated for 14-day and 28-day mortality, as shown in Fig. S2. For each patient, a higher total score indicated a lower probability of 90-day survival.

Scores were assigned for serum phosphate level, CCI, MAP, levels of BUN, Cr and ALB, and SOFA by drawing a line upward from the corresponding values to the ‘score’ line. The sum of all these scores, plotted on the ‘Total score’ line, corresponds to predictions of 90-day survival probability in ICU patients with AKI undergoing CRRT.

Based on the nomogram, predictor lines were drawn upward to confirm these points. The sum of these points was located on the ‘Total Points’ axis. A vertical line was drawn on the bottom scale to determine the likelihood of survival after 90 days. After the development of the prediction model, internal validation was conducted using data from the validation set.

### Predictive accuracy and net benefit of the nomogram

**Fig. 3:**
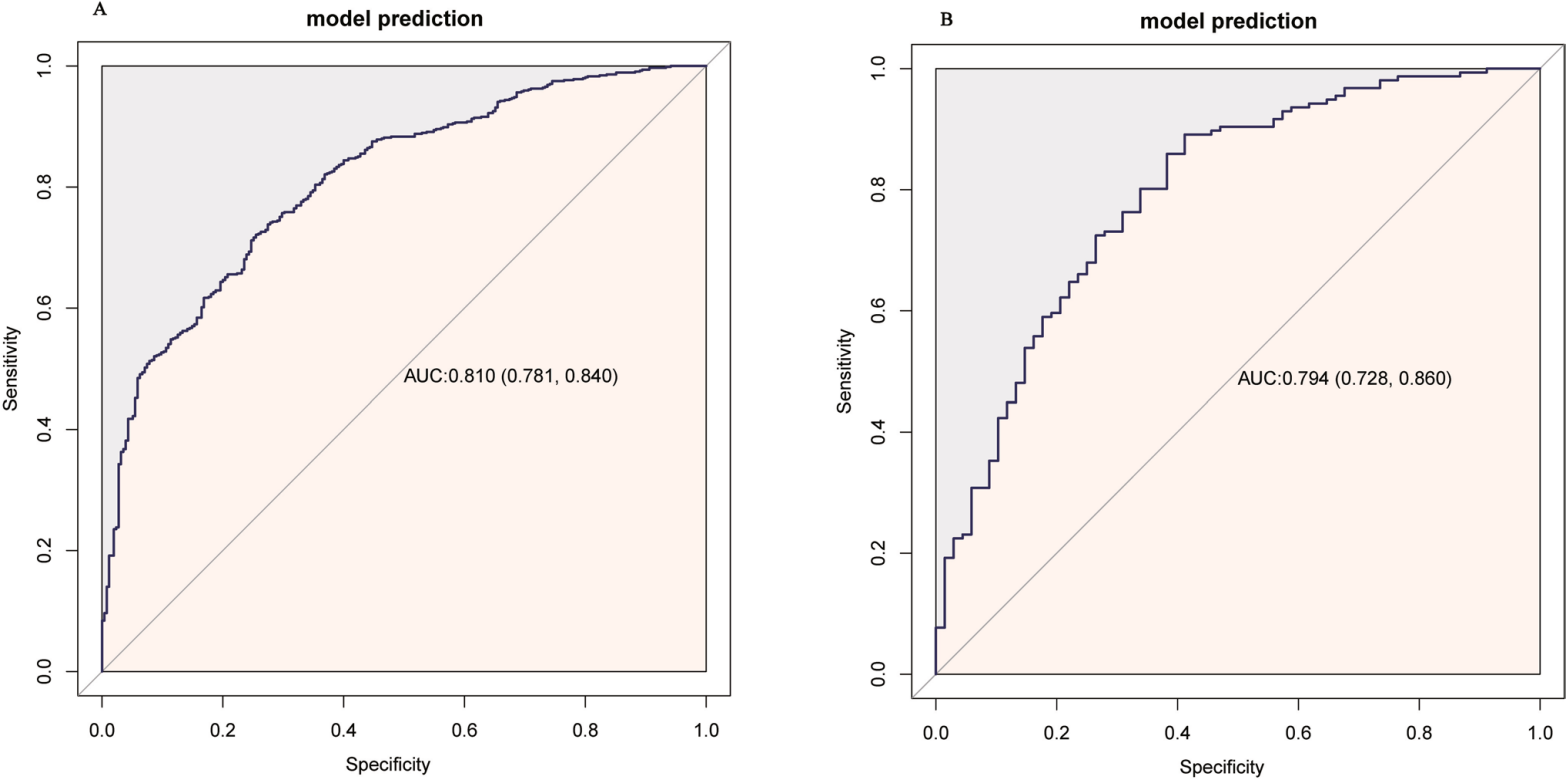
ROC curve and AUC of the nomogram for 90-day mortality in the training set (**A**) and validation set (**B**). *Abbreviations*: ROC, receiver operating characteristic; AUC, area under the ROC curve

**Fig. 4:**
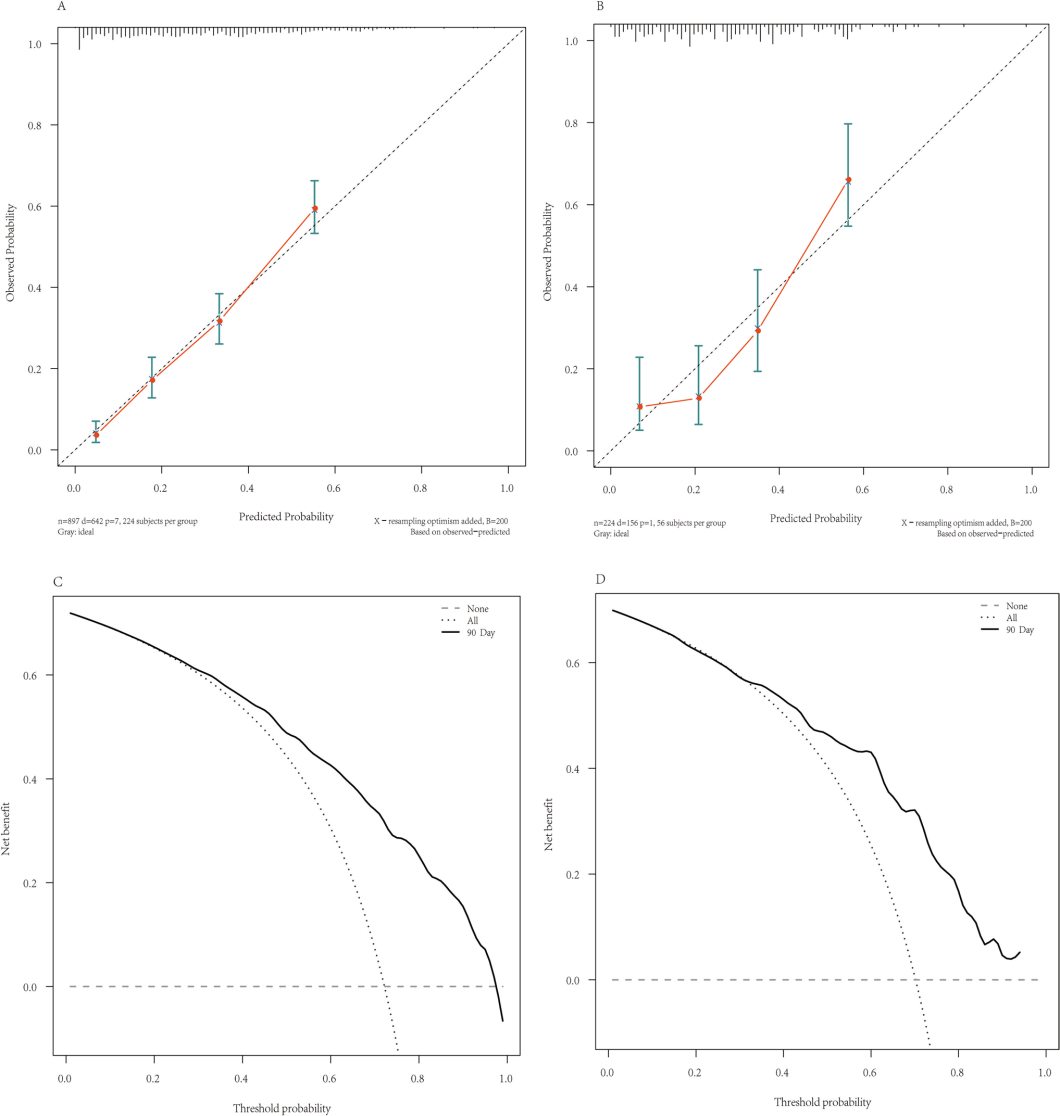
Calibration curves (**A**: Training set; **B**: Validation set) and Decision curves (**C**: Training set; **D**: Validation set) to predict 90-day mortality. The x-axis of the calibration curves represents the predicted probability calculated by the nomogram, and the y-axis is the observed actual probability of 90-day mortality. The clinodiagonal represents a perfect prediction by an ideal model. Decision curves showing the clinical usefulness of the Nomogram prediction model. The abscissa represents threshold probability, the ordinate represents net benefit for patients. The horizontal line (“None”) represents no clinical benefit for all patients without prediction and intervention. The gray line (“All”) represents the clinical benefit of intervention for all patients, and the black curve represents the clinical benefit of using the nomogram prediction model

The training set had an AUC of 0.810 (95% CI: 0.781–0.840, *p* < 0.001) (Fig. 3A), and the calibration curve was close to the ideal diagonal line (Fig. 4A). Furthermore, decision curve analysis showed a significantly better net benefit in the predictive model (Fig. 4C).

The nomogram was internally validated using 224 patients from the validation set. The AUC value was 0.794 (95% CI: 0.728–0.860, *p* < 0.001) (Fig. 3B), indicating the high accuracy of the nomogram. The calibration curve was close to the ideal diagonal line, indicating the good consistency of the model (Fig. 4B). Furthermore, the decision curve analysis demonstrated significant net benefits for both the predictive model and the validation set (Fig. 4D). Based on these data, we conclude that our prediction model can contribute significantly to clinical decision-making. Additionally, we evaluated the model's predictive accuracy for 28-day mortality and found it to be as effective as for 90-day mortality. Details are presented in Supplementary Fig. S3 and S4.

## Discussion

In a cohort of critically ill patients with AKI, those requiring CRRT had 90-day mortality risks of 71.6% and 69.6% in the training and validation sets, respectively. Given the high mortality rate among these patients, it would be beneficial to develop better predictive tools to help reduce long-term mortality rates. In the past few years, researchers have attempted to optimize prediction models for patients with AKI. Nevertheless, existing models either demonstrate limited predictive efficacy for assessing the prognosis of patients undergoing CRRT [15] or they fail to specifically address patients initiating CRRT for AKI [8, 9]. While some recent studies have identified risk factors associated with mortality in CRRT-treated patients [16, 17], the current utility of these variables in accurately predicting mortality within clinical practice remains insufficient. Consequently, there is a need to develop a multivariate mortality prediction model since a single clinical variable may not be sufficient to accurately predict patient outcomes.

In this study, we developed a simple and rapid risk model for predicting the 90-day mortality in critically ill patients with AKI undergoing CRRT. Our prediction model demonstrated robust discrimination and calibration across both the training and validation sets, addressing the notable absence of reliable predictive models in the ICU setting, particularly given the high prevalence of AKI. The predicted probability of 90-day mortality based on the regression coefficient can be calculated using the formula, where LP (linear predictor) is equal to 0.132 × P_24h + 0.069 × CCI − 0.009 × MAP + 0.005 × BUN − 0.225 × Cr − 0.415 × ALB + 0.103 × SOFA. Here, we described the use of the nomogram model. For example, we assumed that the patient had a P_24 h level of 4 mg/dL, CCI score of 4, MAP level of 60 mmHg, Cr level of 8 mg/dL, an ALB level of 2.5 mg/dL, BUN level of 8 mg/dL, and a SOFA score of 14. As shown in Fig. 2, the score corresponding to each parameter was obtained from the first row (the “Point” axis). Finally, the overall score was calculated as the sum of the points for all parameters [15 (P_24 h) + 8 (CCI) + 17.5 (MAP) + 50 (Cr) + 40 (ALB) + 15 (BUN) + 40 (SOFA) = 185.5]. In this scenario, there is an approximately 45% chance of survival for 90 days.

Using this predictive model, we identified several main factors associated with mortality within 90 days of CRRT initiation. AKI is usually diagnosed based on BUN and serum Cr measurements. However, it is neither sensitive nor suboptimal for the diagnosis of AKI. Therefore, there is a need to explore other outcome predictors in critically ill patients with AKI treated with CRRT. In our study, the SOFA score and CCI at CRRT initiation were associated with an increased mortality risk, which is consistent with previous studies [18–20]. Our results are also in agreement with the findings of Jung et al. [12] who showed that among critically ill patients, phosphate is a good biomarker of disease severity and can predict adverse outcomes. Based on our findings, residual hyperphosphatemia remains a prognostic factor. The survival rate of patients with reduced phosphate levels at 24 h after CRRT initiation was significantly higher than those with stable or elevated levels. Interestingly, we found that higher serum creatinine concentrations were associated with a lower risk of mortality within 90 days of CRRT initiation, which was confirmed in many other studies [21–24]. The correlation between elevated serum creatinine levels and improved survival may stem from factors such as enhanced nutritional status, reduced volume overload, or pre-existing CKD [24]. Regarding MAP and ALB, our nomogram showed that the lower the MAP and ALB at the time of CRRT initiation, the greater the mortality risk. Previous studies [25] have shown that a low MAP at CRRT initiation is associated with a high risk of mortality, particularly when it is < 82.7 mmHg. This value can be used for risk classification and as a potential therapeutic target. In another study [26], Kim et al. evaluated ALB levels as a significant and independent prognostic factor for death at 28 and 90 days among patients with sepsis and AKI undergoing CRRT.

Our study highlighted the importance of early identification of high-risk AKI patients to implement targeted therapies that may improve survival rates. By identifying patients with higher SOFA scores, elevated serum creatinine levels, and other poor prognosis-associated risk factors, our model promotes preemptive clinical actions. For example, we can intensify monitoring and interventions for patients with elevated SOFA scores, adjust renal support strategies based on serum creatinine levels and other renal function markers, and implement comprehensive management plans for patients with a high Charlson Comorbidity Index, addressing their multiple comorbidities. These proactive measures underscore our commitment to not only predicting outcomes but also to informing clinical practices that can lead to tangible improvements in patient care and survival.

The inclusion of the 28-day mortality analysis demonstrated trends consistent with the primary endpoint of 90-day mortality, further validating the stability and reliability of our prediction model. While the 28-day mortality reflects short-term outcomes, the 90-day mortality provides a broader perspective on long-term prognosis, making it a more comprehensive endpoint for critically ill patients undergoing CRRT. Our model exhibited good predictive performance at both time points, emphasizing its flexibility and effectiveness in clinical decision-making. This dual assessment at 28 days and 90 days not only reinforces the robustness of our model but also offers clinicians a nuanced understanding of patient risk at different stages of recovery.

Our study has several advantages. First, the variables incorporated in the predictive model are easy to obtain in the clinical setting and can reflect the disease activity of patients, thereby providing clinically relevant information to help identify patients with AKI at a high risk of mortality within the population of patients with AKI requiring CRRT. Second, the potential impact of missing data on our results was assessed using multiple imputations, as shown in Table S2. According to multiple imputations, the data were missing at random and did not show any significant biases. Moreover, we internally validated the prediction model for 90-day mortality.

Our study has some limitations. First, due to its observational, retrospective, single-center design, generalizability and control over confounding factors are limited. We advise caution in broadly applying our results and suggest future multicenter studies for validation. Despite these limitations, our analysis provides valuable insights into CRRT mortality predictors. Second, this was a single center, retrospective study, and some data were missing. We supplemented the data through multiple imputation functions of statistical software to reduce the bias of research results. Third, our analysis relied on pre-existing data from an online repository, which did not include key variables such as “AKI duration,” “the number of days on CRRT,” and “AKI staging according to KDIGO criteria,” nor did it include “ICU length of stay,” “post-ICU hemodialysis requirements,” and “recovery rates.” These variables, as highlighted in the study by Peerapornratana et al. [27] and Sean M. Bagshaw et al [28], are crucial for a comprehensive assessment of patient outcomes and survival post-AKI. Although their absence limits our analysis, our results still contribute meaningfully to the understanding of CRRT mortality predictors. We will strive to include these variables in future studies to offer a more holistic view of patient outcomes. Lastly, the prediction model was not externally validated. In the future, studies should be conducted to externally validate the performance of our model using our databases.

## Conclusions

In conclusion, we developed and internally validated a model for predicting 90-day mortality in ICU patients with AKI undergoing CRRT, using objective data routinely collected in clinical practice. These included the SOFA score; levels of serum Cr, BUN, and ALB; CCI; MAP at CRRT initiation; and P_24 h. This predictive model performed well and may be helpful in risk stratification and decision-making in clinical situations.

## Supplementary Information

Below is the link to the electronic supplementary material.

Supplementary file1 (DOCX 3399 KB)

## Data Availability

The datasets used and/or analyzed in the current study are available from 10.5061/dryad.6v0j9.
