# Supplementary material for: Development and validation of a prediction model for 90-day mortality among critically ill patients with AKI undergoing CRRT
Source: J Nephrol. 2025 Mar 10;38(3):947–57. doi: 10.1007/s40620-025-02237-1 (PMC12165874; doi:10.1007/s40620-025-02237-1)
Supplement: Supplementary file 1 — Supplementary file1 (DOCX 3399 KB) [file 40620_2025_2237_MOESM1_ESM.docx]

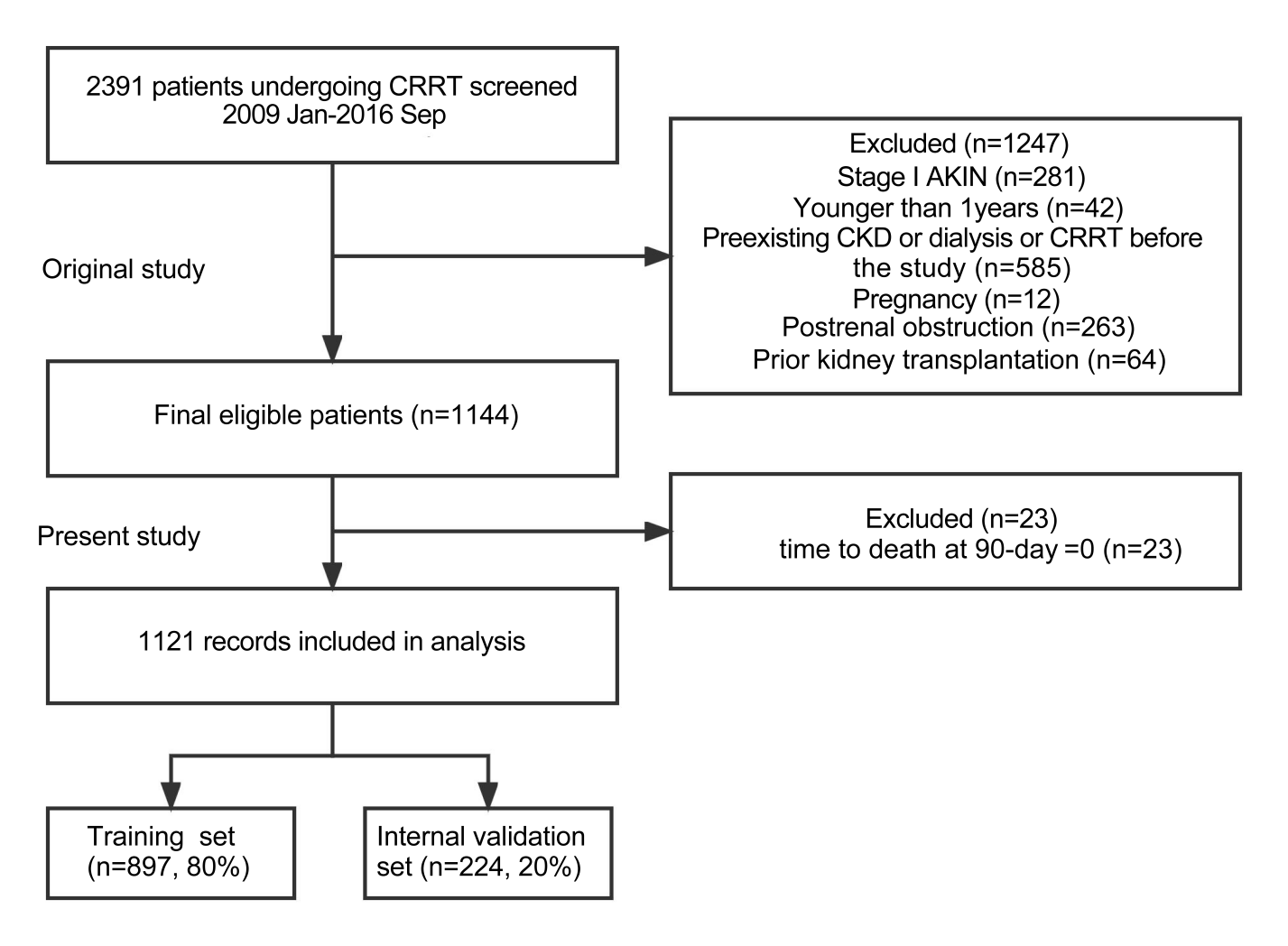


**Figure S1. Flowchart of the patient selection process.**

***Abbreviations:*** CRRT, continuous renal replacement therapy; AKIN, acute kidney injury network; CKD, chronic kidney disease


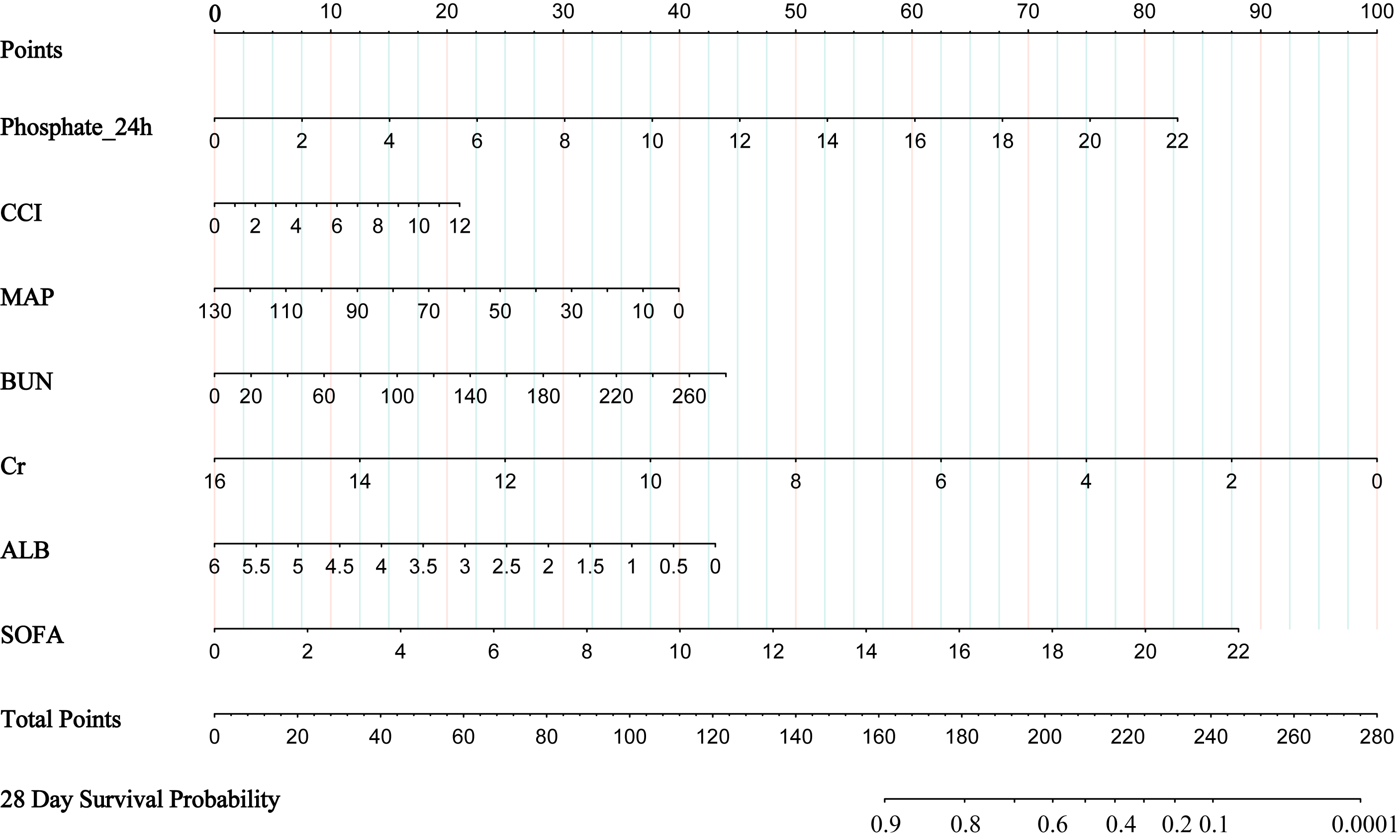


**Figure S2.** Nomogram to calculate risk score and predict the risk of 14, 28, and 90-day mortality

***Abbreviations:*** Phosphate_24 h, serum phosphate at 24 hours after CRRT initiation; CCI, charlson comorbidity index; MAP, mean arterial pressure; BUN, blood urea nitrogen; Cr, Creatinine; ALB, albumin; SOFA, sequential organ failure assessment


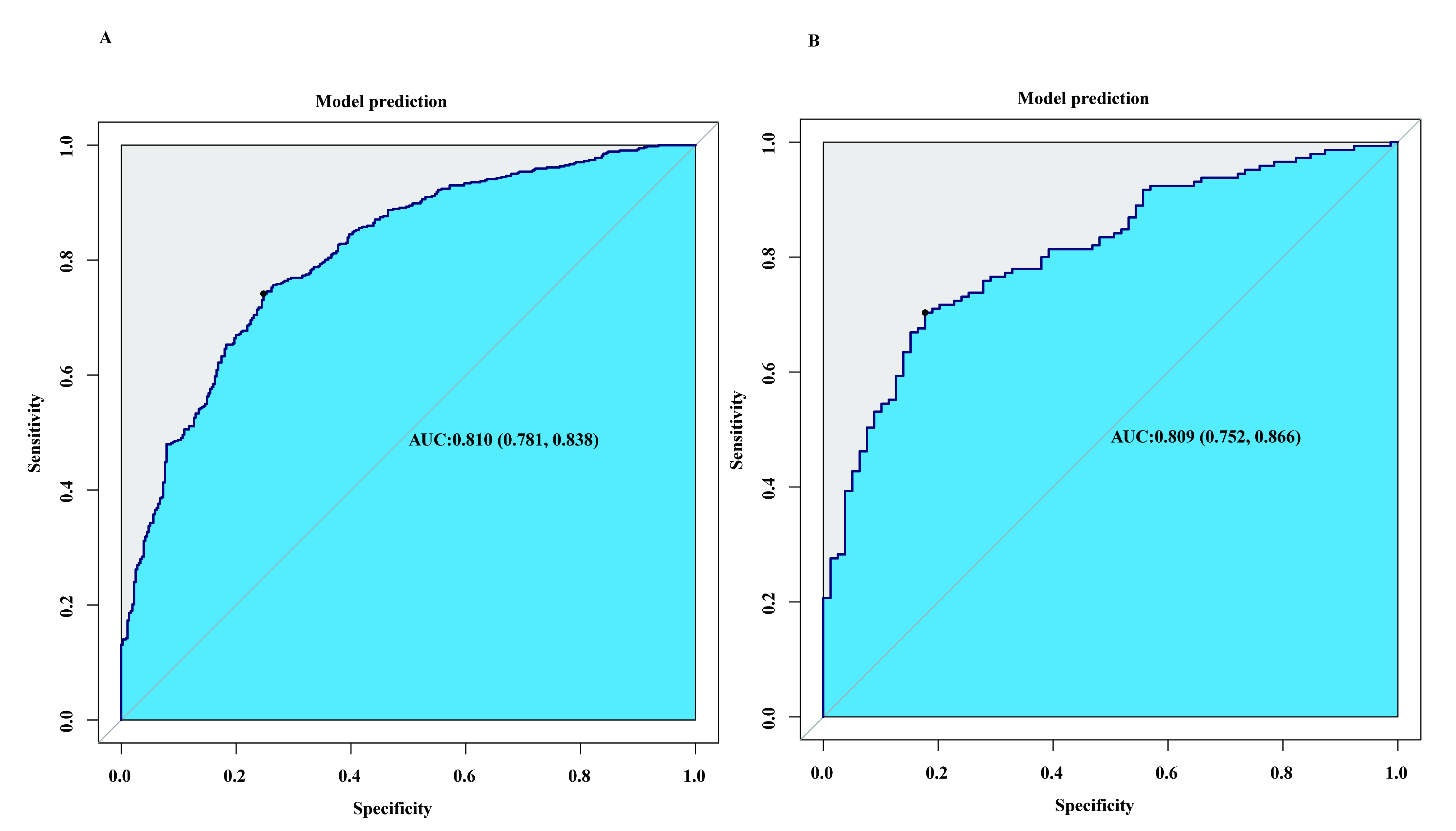


**Figure S3.** ROC curve and AUC of the nomogram for 28-day mortality in the training set (**A**) and validation set (**B**).

***Abbreviations:*** ROC, receiver operating characteristic; AUC, area under the ROC curve


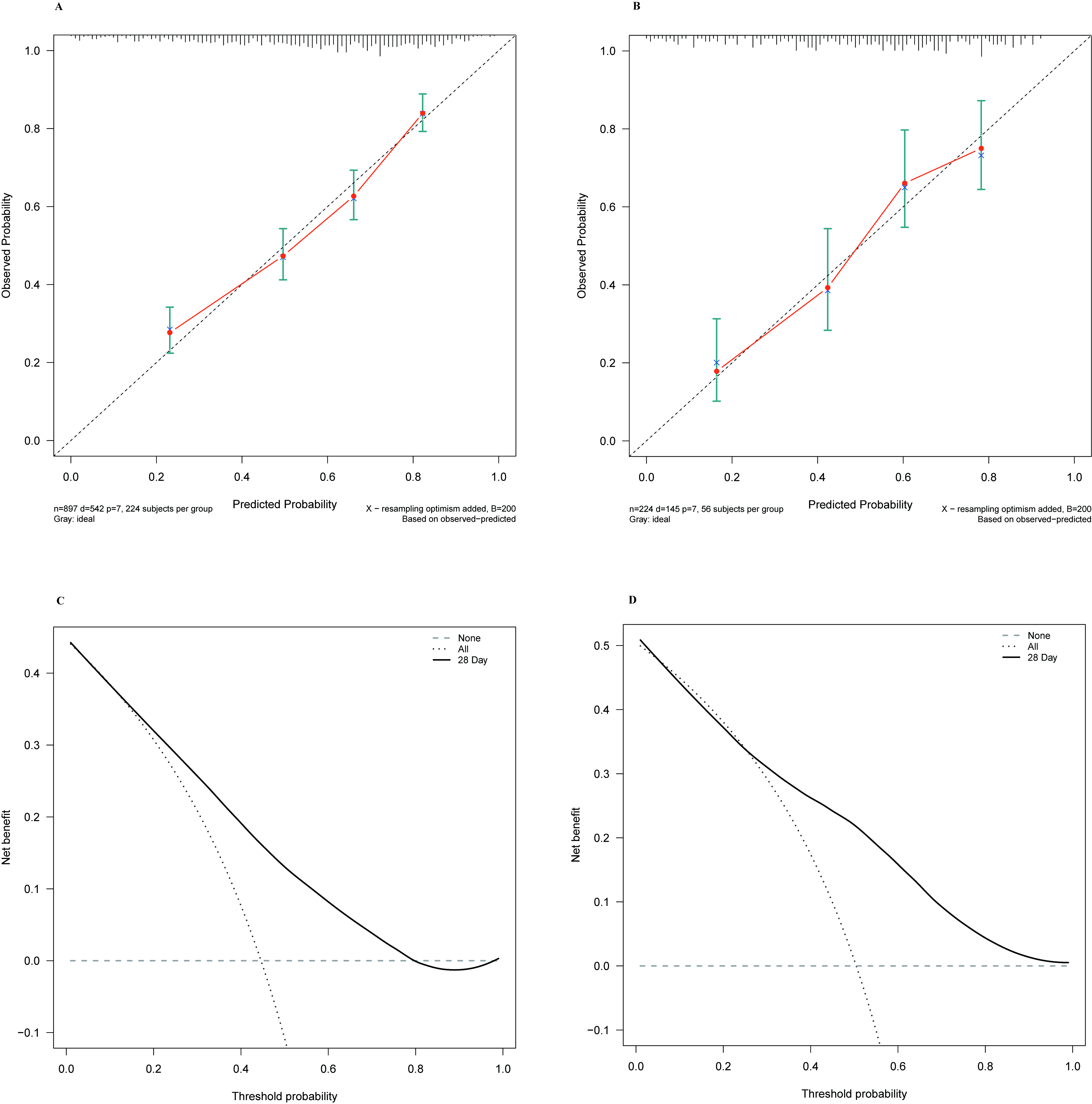


**Figure S4.** Calibration curves ( A: Training set; B: Validation set) and Decision curves ( C: Training set; D: Validation set) to predict 28-day mortality. The x-axis of the calibration curves represents the predicted probability calculated by the nomogram, and the y-axis is the observed actual probability of 28-day mortality. The clinodiagonal represents a perfect prediction by an ideal model. Decision curves shows the clinical usefulness of the Nomogram prediction model. The abscissa represents threshold probability, the ordinate represents net benefit for patients. The horizontal line (“None”) represents no clinical benefit for all patients without prediction and intervention. The gray line (“All”) represents the clinical benefit of intervention for all patients, and the black curve represents the clinical benefit of using the nomogram prediction model.


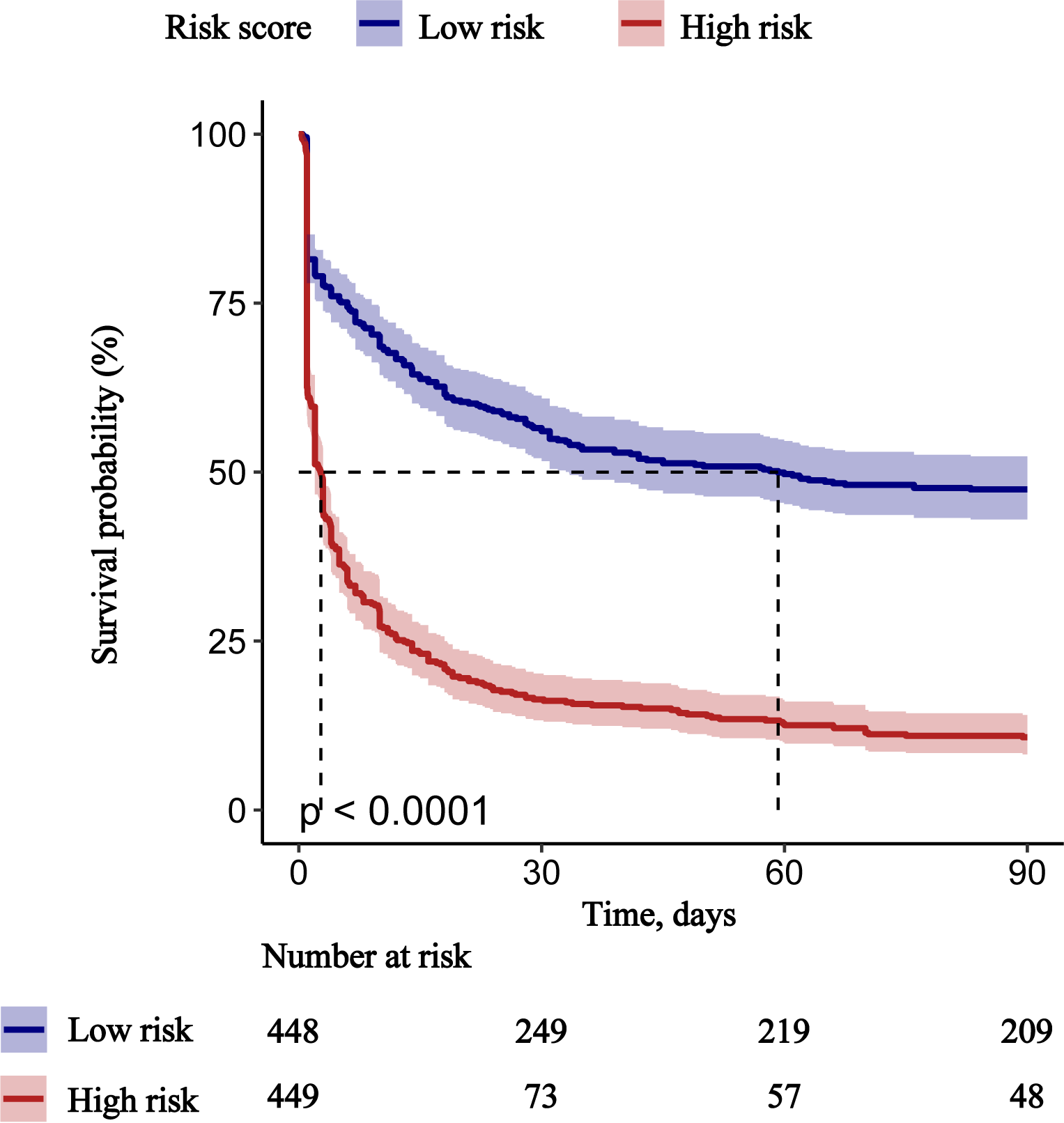


**Figure S5.** Kaplan-Meier survival curves of 90-day mortality.


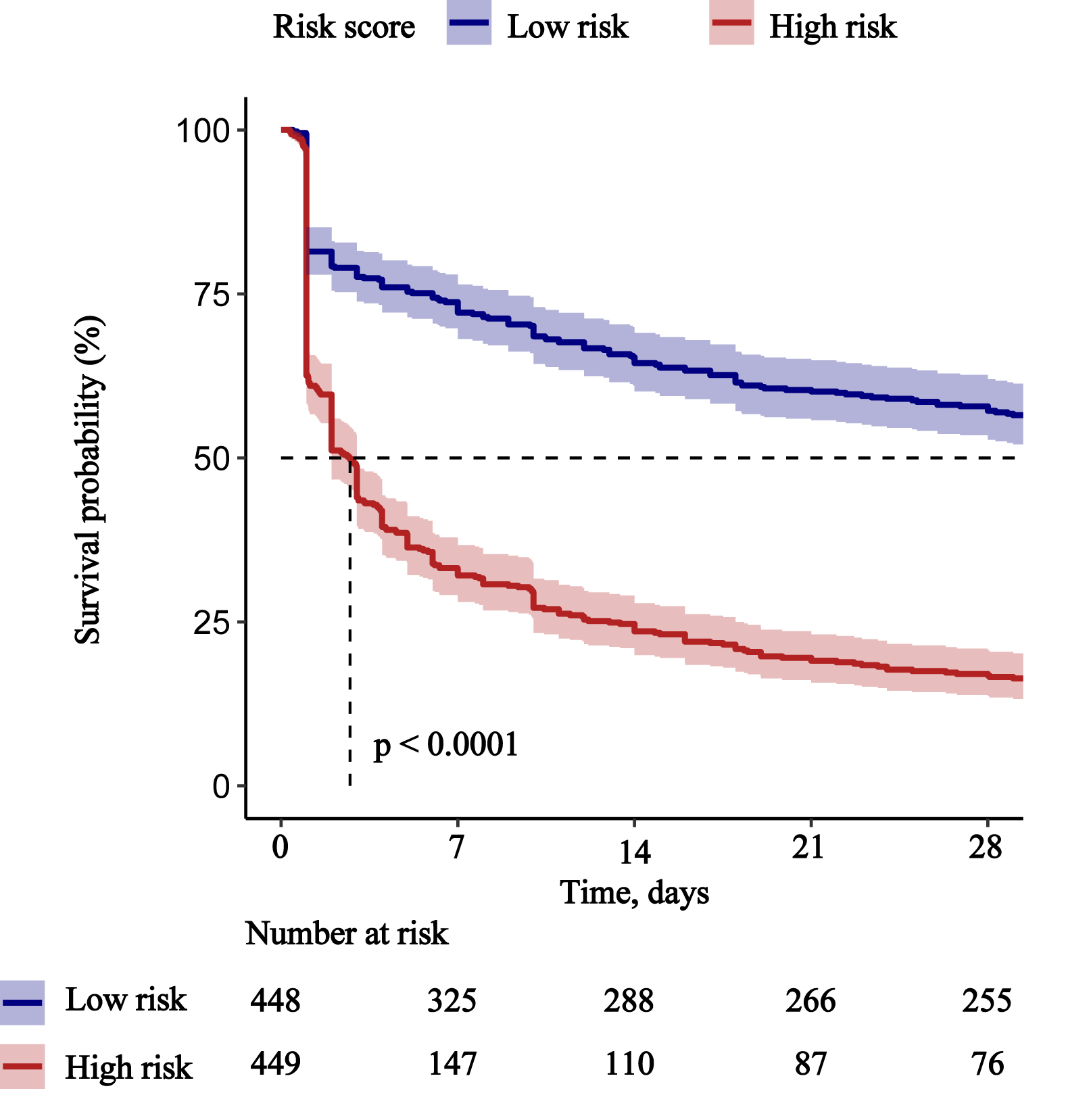


**Figure S6.** Kaplan-Meier survival curves of 28-day mortality.


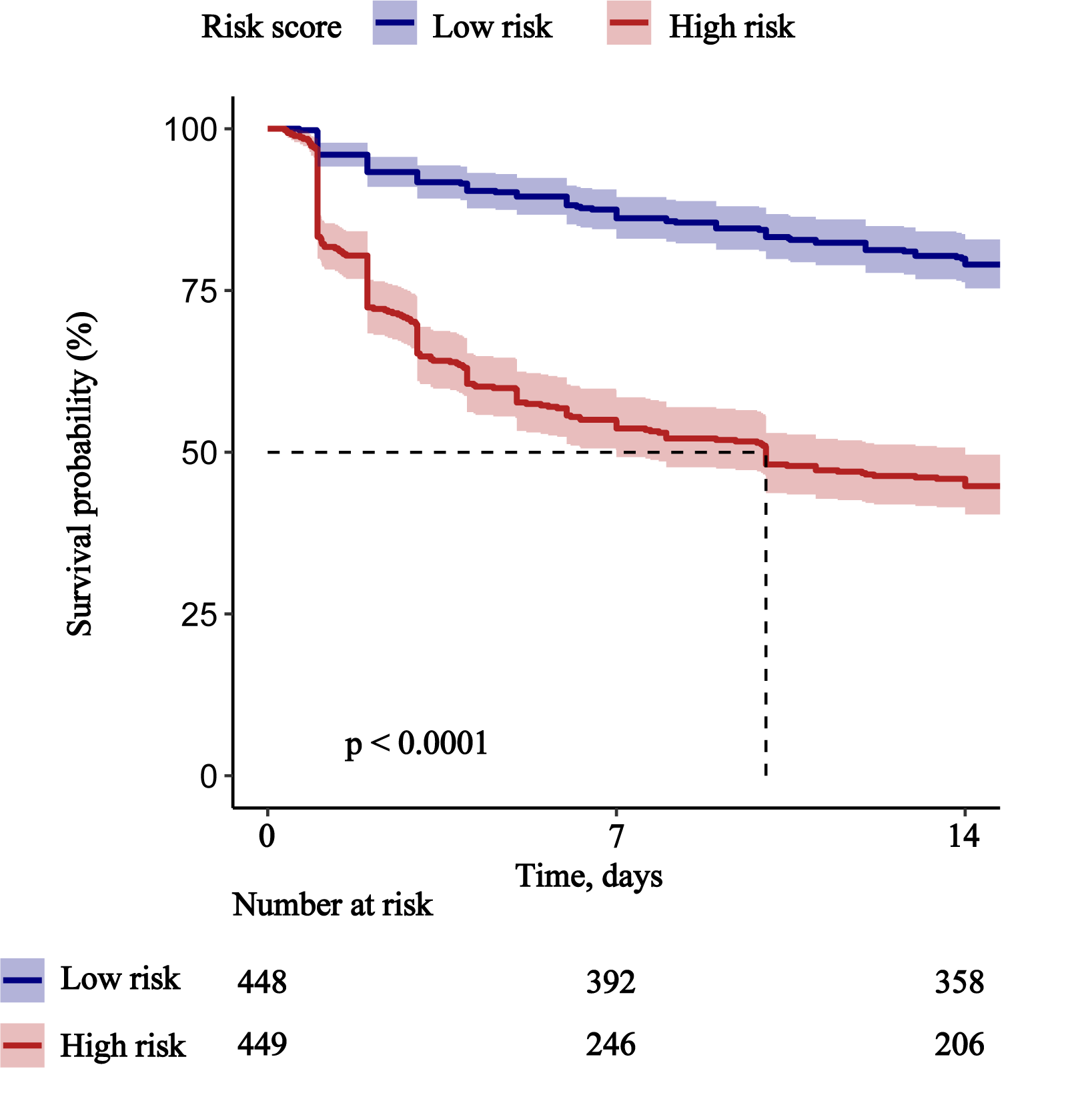


**Figure S7.** Kaplan-Meier survival curves of 14-day mortality.

**Table S1.** Sensitivity analysis results before and after multiple imputations

| **Variables** | **Unit** | **Number（%）with missing data** | **Complete case** |  | **Multiple imputation** |
| --- | --- | --- | --- | --- | --- |
|  |  |  | **HR (95% CI)** |  | **HR (95% CI)** |
| Potassium | mEq/L | 12(1.0) | 0.93 (0.82-1.06) |  | 1.04 (0.96-1.13) |
| Bicarbonate | mEq/L | 128(11.2) | 1.00 (0.97-1.03) |  | 1.00 (0.98-1.01) |
| P_0h | mg/dL | 65(5.8) | 1.19 (0.82-1.73) |  | 1.05 (0.73-1.49) |
| P_24h | mg/dL | 138(12.3) | 0.98 (0.68-1.39) |  | 1.07 (0.77-1.50) |
| Delta_P | mg/dL | 157(14.0) | 1.17 (0.8-1.72) |  | 1.06 (0.74-1.51) |
| PreCr | mg/dL | 488(42.7) | 1 (0.98-1.02) |  | 0.99 (0.98-1.01) |
| Cr | mg/dL | 6(0.5) | 0.73 (0.61-0.87) |  | 0.81 (0.74-0.88) |
| BMI | kg/m2 | 20(1.8) | 0.99 (0.97-1.02) |  | 0.98 (0.97-1) |
| SBP | mmHg | 2(0.2) | 0.99 (0.96-1.02) |  | 0.99 (0.99-1) |
| DBP | mmHg | 2(0.2) | 1 (0.95-1.06) |  | 1 (1-1.01) |
| MV | % | 1(0.1) | 0.73 (0.49-1.09) |  | 0.84 (0.66-1.06) |
| WBC | μL | 10(0.9) | 1 (1-1) |  | 1 (1-1) |
| Hb | g/dL | 4(0.3) | 0.98 (0.93-1.04) |  | 0.98 (0.95-1.02) |
| BUN | mg/dL | 6(0.5) | 1.01 (1-1.01) |  | 1 (1-1.01) |
| ALB | g/dL | 10(0.9) | 0.77 (0.6-1) |  | 0.65 (0.57-0.74) |
| CRP | mg/L | 275(24.0) | 1 (1-1) |  | 1 (1-1) |
| UO | ml | 6(0.5) | 1 (1-1) |  | 1 (1-1) |
| APACHE II |  | 13(1.1) | 1.01 (1-1.03) |  | 1.01 (1-1.02) |
| SOFA |  | 3(0.3) | 1.11 (1.06-1.16) |  | 1.12 (1.09-1.15) |

***Abbreviations:*** P_0h, phosphate at CRRT initiation; P_24h, phosphate at 24 h after CRRT initiation; Delta_p, phosphate change(P_24h - P_0h); PreCr, creatinine level before CRRT initiation; Cr, Creatinine level at CRRT initiation; BMI, Body mass index; SBP, Systolic blood pressure; DBP, Diastolic blood pressure; MV, Mechanical ventilation; WBC, White blood cell; Hb*,* Hemoglobin; BUN, Blood urea nitrogen; ALB, albumin; CRP, C-reactive protein; UO, urine output at 2 h after CRRT initiation; APACHE II, Acute Physiology and Chronic Health Evaluation II; SOFA, Sequential Organ Failure Assessment.

**Table S2.** Comparison of Baseline Patient Characteristics Between 90 day-survivors and non-survivors and Between Training and Validation Sets

| **Variables** | **Training set** | | ***P*-value** | **Validation set** | | ***P*-value** |
| --- | --- | --- | --- | --- | --- | --- |
|  | **Survivors**  **(n = 255)** | **non-survivors**  **(n = 642)** |  | **Survivors**  **(n = 68)** | **non-survivors**  **(n = 156)** |  |
| Age (years) | 63.2 ± 14.8 | 63.8 ± 14.1 | 0.625 | 60.2 ± 16.2 | 62.9 ± 13.5 | 0.183 |
| Men (%) | 150 (58.8) | 395 (61.5) | 0.455 | 46 (67.6) | 98 (62.8) | 0.488 |
| HTN (%) | 163 (63.9) | 313 (48.8) | <0.001 | 41 (60.3) | 75 (48.1) | 0.092 |
| DM (%) | 106 (41.6) | 212 (33) | 0.03 | 25 (36.8) | 48 (30.8) | 0.379 |
| MI (%) | 29 (11.4) | 64 (10) | 0.534 | 7 (10.3) | 11 (7.1) | 0.412 |
| HF (%) | 49 (19.2) | 105 (16.4) | 0.305 | 9 (13.2) | 23 (14.7) | 0.767 |
| CVD (%) | 30 (11.8) | 61 (9.5) | 0.576 | 7 (10.3) | 13 (8.3) | 0.636 |
| COPD (%) | 22 (8.6) | 41 (6.4) | 0.236 | 7 (10.3) | 9 (5.8) | 0.262 |
| MV (%) | 168 (65.9) | 530 (82.6) | <0.001 | 49 (72.1) | 130 (83.3) | 0.053 |
| CCI | 2.0 (1.0, 3.5) | 3.0 (2.0, 5.0) | <0.001 | 2.0 (1.0, 4.2) | 3.0 (2.0, 5.0) | 0.094 |
| Cause of AKI |  |  | 0.032 |  |  | 0.796 |
| Sepsis | 167 (65.5) | 467 (72.7) |  | 46 (67.6) | 110 (70.5) |  |
| Nephrotoxin | 12 (4.7) | 17 (2.6) |  | 1 (1.5) | 5 (3.2) |  |
| Ischemia | 20 (7.8) | 56 (8.7) |  | 6 (8.8) | 9 (5.8) |  |
| Surgery | 31 (12.2) | 43 (6.7) |  | 5 (7.4) | 14 (9) |  |
| Others | 25 (9.8) | 59 (9.2) |  | 10 (14.7) | 18 (11.5) |  |
| Cause of CRRT |  |  | 0.035 |  |  | 0.712 |
| Volume overload (%) | 46 (18) | 80 (12.5) |  | 9 (13.2) | 19 (12.2) |  |
| Metabolic acidosis (%) | 41 (16.1) | 152 (23.7) |  | 14 (20.6) | 30 (19.2) |  |
| Hyperkalemia (%) | 10 (3.9) | 38 (5.9) |  | 2 (2.9) | 5 (3.2) |  |
| Uremia (%) | 26 (10.2) | 58 (9) |  | 11 (16.2) | 18 (11.5) |  |
| Oliguria (%) | 74 (29) | 159 (24.8) |  | 19 (27.9) | 39 (25) |  |
| Others (%) | 58 (22.7) | 155 (24.1) |  | 13 (19.1) | 45 (28.8) |  |
| AKIN stages |  |  | 0.948 |  |  | 0.716 |
| Stage 2 (%) | 65 (25.5) | 165 (25.7) |  | 18 (26.5) | 45 (28.8) |  |
| Stage 3 (%) | 190 (74.5) | 477 (74.3) |  | 50 (73.5) | 111 (71.2) |  |
| BMI (kg/m^2^) | 24.2 ± 4.1 | 23.6 ± 4.8 | 0.092 | 24.8 ± 4.9 | 23.2 ± 3.6 | 0.006 |
| SOFA | 10.0 ± 3.4 | 12.8 ± 3.3 | <0.001 | 10.6 ± 3.4 | 12.9 ± 3.2 | < 0.001 |
| APACHE II | 24.7 ± 7.4 | 28.4 ± 7.9 | <0.001 | 25.9 ± 9.3 | 27.0 ± 7.3 | 0.342 |
| SBP (mmHg) | 117.2 ± 21.4 | 109.4 ± 20.6 | <0.001 | 121.1 ± 21.7 | 112.0 ± 18.3 | 0.002 |
| DBP (mmHg) | 62.0 ± 14.3 | 59.4 ± 14.1 | 0.014 | 65.4 ± 15.5 | 59.8 ± 13.0 | 0.005 |
| MAP (mmHg) | 80.4 ± 15.1 | 75.9 ± 14.0 | <0.001 | 84.5 ± 15.3 | 77.2 ± 13.5 | < 0.001 |
| Hemoglobin (g/dL) | 10.0 ± 2.2 | 9.5 ± 2.2 | <0.001 | 10.0 ± 2.7 | 9.6 ± 2.0 | 0.216 |
| WBC (μL) | 13660.0 (9130.0, 20400.0) | 11220.0 (5478.0, 17940.0) | <0.001 | 12150.0 (9005.0, 19140.0) | 9740.0 (4572.0, 18740.0) | 0.049 |
| Albumin (g/dL) | 2.8 ± 0.6 | 2.6 ± 0.5 | <0.001 | 2.8 ± 0.6 | 2.5 ± 0.6 | < 0.001 |
| Potassium (mEq/L) | 4.7 ± 1.1 | 4.8 ± 1.1 | 0.344 | 4.7 ± 1.0 | 4.5 ± 1.0 | 0.153 |
| Bicarbonate (mEq/L) | 17.0 ± 5.3 | 16.9 ± 5.8 | 0.773 | 16.8 ± 4.9 | 17.0 ± 6.1 | 0.791 |
| BUN (mg/dL) | 46.0 (31.0, 66.5) | 52.0 (34.0, 76.8) | 0.008 | 46.0 (35.0, 60.0) | 55.0 (34.8, 75.2) | 0.198 |
| Phosphate (mg/dL) | 5.3 ± 2.4 | 6.0 ± 2.5 | <0.001 | 5.4 ± 2.0 | 5.7 ± 2.2 | 0.323 |
| Creatinine (mg/dL) | 3.0 ± 2.0 | 2.6 ± 1.4 | <0.001 | 3.2 ± 2.2 | 2.6 ± 1.8 | 0.027 |
| CRRT dose (ml/kg) | 36.2 ± 4.7 | 36.9 ± 4.8 | 0.034 | 36.7 ± 5.9 | 36.3 ± 4.2 | 0.57 |
| CRP (mg/L) | 66.7 (18.9, 153.2) | 73.6 (20.8, 172.0) | 0.19 | 66.8 (23.6, 142.9) | 100.0 (30.7, 197.3) | 0.053 |

***Abbreviations:*** HTN, hypertension; DM, diabetes mellitus; MI, myocardial infarction; HF, heart failure; CVD, cerebrovascular disease; COPD, chronic obstructive pulmonary disease; MV, mechanical ventilation; CCI, charlson comorbidity index; AKI, acute kidney injury; CRRT, continuous renal replacement therapy; AKIN, acute kidney injury network; BMI, body mass index; SOFA, sequential organ failure assessment; APACHE II, acute physiology and chronic health evaluation II; SBP, systolic blood pressure; DBP, diastolic blood pressure; MAP, mean arterial pressure; WBC, white blood cells; BUN, blood urea nitrogen; CRP, C-reactive protein.***Abbreviations:*** HTN, hypertension; DM, diabetes mellitus; MI, myocardial infarction; HF, heart failure; CVD, cerebrovascular disease; COPD, chronic obstructive pulmonary disease; MV, mechanical ventilation; CCI, charlson comorbidity index; AKI, acute kidney injury; CRRT, continuous renal replacement therapy; AKIN, acute kidney injury network; BMI, body mass index; SOFA, sequential organ failure assessment; APACHE II, acute physiology and chronic health evaluation II; SBP, systolic blood pressure; DBP, diastolic blood pressure; MAP, mean arterial pressure; WBC, white blood cells; BUN, blood urea nitrogen; CRP, C-reactive protein
